# Editorial: Spotlight on Solanaceae Metabolism: Biotechnological Application

**DOI:** 10.3389/fpls.2021.756948

**Published:** 2021-09-17

**Authors:** Zsófia Bánfalvi, Amalia Barone, Glenn J. Bryan

**Affiliations:** ^1^National Agricultural Research and Innovation Centre, Gödöllő, Hungary; ^2^Department of Agricultural Sciences, University of Naples Federico II, Naples, Italy; ^3^Cell and Molecular Sciences, The James Hutton Institute, Dundee, United Kingdom

**Keywords:** Solanaceae, transcriptomics, metabolomics, stress tolerance, nutritional quality, tomato flavor, eggplant browning, potato drought and heat tolerance

The Solanaceae family consists of more than 90 genera and nearly 3,000 species distributed throughout the world with a great diversity, and contains many species with economic importance as food and medicinal plants, spices and ornamentals. Among the most important agricultural crops are potato, tomato, pepper, eggplant and tobacco, which serve also as model systems for the investigation of fundamental biological and agronomic questions. The recently developed “omics” technologies and their various combinations have started to shed light on flower, fruit and tuber development, as well as on abiotic and biotic stress responses of sensitive and stress-tolerant varieties, and provided molecular markers for breeders. In this Research Topic, we provided a platform for articles based on “omics” studies in Solanaceae plants to further advance our knowledge on the perturbation of metabolic pathways in different conditions, and their regulation for improved biosynthesis of molecules of interest.

Global climate change with rising temperatures and reduced water availability is challenging agricultural systems worldwide. Potato plants are very sensitive to drought and elevated temperatures. To improve water use efficiency as well as tuberization of potato plants under drought and heat stress Lehretz et al. co-expressed *HEXOKINASE 1* from *Arabidopsis thaliana* (*AtHXK1*) and *SELF-PRUNING 6A* (*SP6A*) from *Solanum tuberosum*, encoding a mobile tuberisation signal, in guard cells and shoots, respectively. Although the plants showed a bushy growth habit the yield and starch content of tubers were only slightly affected by the heat and drought stress combination. Dehydration-responsive element binding (DREB) transcription factors play crucial regulatory roles in stress responses. Mao et al. found that the only *DREB* that responded to heat was *SlDREBA4* in the model tomato cultivar Microtom. Overexpression of *SlDREBA4* enhanced tolerance of tomato plants to heat stress by regulating the expression of many heat shock- and calcium-binding protein genes, and by altering the content of osmolytes and stress hormones as well as the activities of antioxidant enzymes. It was also demonstrated that application of exogenous ascorbic acid counteracts the salt-induced photoinhibition in tomato (Chen et al.). Thus, these studies provided new genetic resources and rationales for abiotic stress-tolerance breeding of Solanaceae plants.

Plants are confronted not only with abiotic, but also with biotic stresses. *Phytophthora nicotianae* is a soil borne pathogen causing black shank disease in tobacco. Plants have evolved complex defense systems against pathogens including root exudates of some resistant varieties with an inhibitory effect on pathogen growth. Metabolic profiling performed by Zhang et al. depicted differing metabolic patterns of root exudates between a resistant and susceptible tobacco cultivar. Some of the detected defense compounds (e.g., tartaric-, ferulic-, lauric acid, and phenylpropanoids) have potential for disease control application.

The greenhouse cultivation of tomatoes involves the application of a considerable amount of fertilizers and produces an accumulation of surplus salts in soil. Kim et al. demonstrated that minimal nutrient and water supplies improves tomato quality in soils with surplus salt. Rosa-Martínez et al. dealt with a similar problem and searched for tomato varieties with improved nitrogen use efficiency to reduce the need for fertilization in line with the sustainable agriculture policies. The “de penjar” tomatoes from the Spanish Mediterranean region were selected for the study because these tomatoes are cultivated under low-input conditions and carry the *alc* mutation, which provides long shelf-life for the fruit. A large amount of variation between the local varieties was observed especially in lycopene content that together with β-carotene presented a strongly significant genotype x nitrogen input interaction. These results revealed the potential of “de penjar” varieties as a genetic resource in breeding for low nitrogen inputs, while keeping the high organoleptic and nutritional quality of traditional tomato cultivars. Tomato flavor, which is determined by the right balance of sugars and organic acids, as well as a range of volatile organic compounds, is obviously a primary focus of tomato breeding. Using newly available high quality genome assemblies, Pereira et al. associated five flavor-controlling genes with trait variation and described the genetic diversity at these loci in a red-fruited tomato clade. The tomato collection used in this study would be an excellent set of material to discover new flavor genes through genetic mapping approaches.

Eggplant, cultivated for over 1,500 years, represents the third most important crop of the Solanaceae family after potato and tomato. Most of its nutritional properties are related to phenolic compounds. However, there is a correlation between the concentration of phenolics and browning of the fruit flesh. The polyphenol oxidases (PPOs) catalyze the oxidation of phenolic compounds leading to browning. Based on this knowledge Maioli et al. generated knock-out mutants in three *PPO* genes of an eggplant cultivar using the CRISPR/Cas9 technology. Upon cutting, edited eggplant fruits showed a reduction in browning. Through this approach it will be possible to develop non-, or reduced browning eggplant varieties that maintain their antioxidant and nutritional properties. Metabolic QTLs controlling fruit nutritional quality were discovered by Sulli et al., who analyzed a population of recombinant inbred lines (RILs), derived from two eggplant lines differing in several key agronomic traits using a combination of untargeted and targeted metabolomics approaches. As a result of this study, candidate genes underlying the nutritional quality traits were identified in eggplant.

Hybrid eggplant seed production needs a reliable male-sterile system. Functional genic male sterility (GMS) based on anther indehiscence, i.e., the anthers do not open to release pollen, exists in eggplant. In order to uncover differences in the anther dehiscence network Wang et al. performed transcriptome analysis to compare a fertile line and a male sterile eggplant line and detected major differences in jasmonic acid signal transduction.

We believe that this collection of publications represents well the current stage of Solanaceae “omics” research and thank the authors and reviewers for their valuable support.

## Author Contributions

ZB, AB, and GB: writing and editing. All authors contributed to the article and approved the submitted version.

## Conflict of Interest

The authors declare that the research was conducted in the absence of any commercial or financial relationships that could be construed as a potential conflict of interest.

## Publisher's Note

All claims expressed in this article are solely those of the authors and do not necessarily represent those of their affiliated organizations, or those of the publisher, the editors and the reviewers. Any product that may be evaluated in this article, or claim that may be made by its manufacturer, is not guaranteed or endorsed by the publisher.

